# Validity of reactive attachment disorder and disinhibited social engagement disorder in adolescence

**DOI:** 10.1007/s00787-019-01456-9

**Published:** 2019-12-12

**Authors:** Astrid R. Seim, Thomas Jozefiak, Lars Wichstrøm, Nanna S. Kayed

**Affiliations:** 1 Division of Mental Healthcare, Department of Children and Youth, St. Olavs Hospital, Trondheim, Norway; 2 Department of Mental Health, Faculty of Medicine and Health Sciences, NTNU Norwegian University of Science and Technology, Trondheim, Norway; 3 Department of Psychology, NTNU, Trondheim, Norway

**Keywords:** Adolescence, Reactive attachment disorder, Disinhibited social engagement disorder, Mental health, Residential youth care, Validity

## Abstract

Although reactive attachment disorder (RAD) and disinhibited social engagement disorder (DSED) are acknowledged as valid disorders in young children, controversy remains regarding their validity in adolescence. An unresolved question is whether symptoms of RAD and DSED are better conceptualized as other psychiatric disorders at this age. All adolescents (*N* = 381; 67% consent; 12–20 years old) living in residential youth care in Norway were interviewed to determine the symptoms and diagnosis of RAD/DSED and other common psychiatric disorders using the Child and Adolescent Psychiatric Assessment (CAPA). The construct validity of RAD and DSED, including structural and discriminant validity, was investigated using confirmatory factor analysis and latent profile analysis. Two-factor models distinguishing between symptoms of RAD and DSED and differentiating these symptoms from the symptoms of other psychiatric disorders revealed better fit than one-factor models. Symptoms of RAD and DSED defined two distinct latent groups in a profile analysis. The prevalence of RAD was 9% (95% CI 6–11%), and the prevalence of DSED was 8% (95% CI 5–11%). RAD and DSED are two distinct latent factors not accounted for by other common psychiatric disorders in adolescence. RAD and DSED are not uncommon among adolescents in residential youth care and therefore warrant easy access to qualified health care and prevention in high-risk groups.

**Electronic supplementary material:**

The online version of this article (10.1007/s00787-019-01456-9) contains supplementary material, which is available to authorized users.

## Introduction

Reactive attachment disorder (RAD) and disinhibited social engagement disorder (DSED) are socially disabling disorders caused by insufficient care, such as social neglect, deprivation or limited opportunity to form stable and selective attachments to caregivers [1]. Although research on these disorders has increased considerably in recent years [2, 3], RAD and DSED remain among the least studied and most poorly understood psychiatric disorders in the Diagnostic and Statistical Manual of Mental Disorders (DSM) [4]. Further, most existing research examines young children, and the question of whether RAD and DSED diagnoses should be reserved for the youngest or whether they also apply to older children and adolescents has been raised [1, 2, 5]. Although there is growing evidence that RAD and DSED symptoms may persist into adolescence and early adulthood [5–9], controversy remains regarding their diagnostic classification. Whereas some denominate RAD and DSED as valid diagnostic constructs in adolescence, others question whether symptoms of RAD and DSED may then be better conceptualized as more common psychiatric disorders [2, 10]. Hitherto, RAD and DSED have been evidenced as distinct from other psychopathology until middle childhood [11–16], but their discriminant validity in adolescence remains unstudied. Clarifying uncertainties regarding the possible existence and appearance of RAD and DSED in adolescence has been deemed a priority [3] and is addressed herein by assessing their construct validity, including discriminant validity.

Further, the controversy of RAD and DSED beyond early childhood relates to differences in study populations and measurement [2]. Longitudinal studies of RAD and DSED primarily examine children exposed to early severe institutional deprivation [6, 7, 17–22], a group having contributed considerably to the DSM criteria of RAD and DSED, but for whom the generalizability to less deprived populations is questioned [6, 23]. No gold standard for assessment in older children exists. Although RAD and DSED are reported in cross-sectional studies of older children without early institutionalization, measurement issues have induced uncertainty as to whether the described RAD and DSED in these children differ exceedingly from the presentation in early institutionalized children, or whether the former also qualify for the disorders as defined by DSM-5 [2, 3]. To clarify this, we investigate whether RAD and DSED can be identified in a high-risk group of adolescents, mainly unexposed to early institutionalization but with likely exposure to early in-family social neglect and pathogenic care, using an in-depth psychiatric interview developed and validated for younger children in accordance with DSM [15, 24].

In DSM-5, RAD is characterized by minimally seeking and responding to comfort, concurrent with limited positive affect, minimal social/emotional responsiveness to others and/or unexplained irritability/sadness/fearfulness when interacting with caregivers [1]. Whereas disordered attachment behaviour is core to RAD, DSED may occur regardless of attachment status [25] and is characterized by socially disinhibited behaviour in unfamiliar settings or in interactions with strangers [1]. RAD and DSED are defined as two separate disorders in DSM-5, whereas their corresponding diagnostic constructs in DSM-IV were categorized as two subtypes (inhibited and disinhibited) of one disorder (reactive attachment disorder) [26]. The separation into two disorders derived from research indicating that although RAD and DSED share an aetiology, they differ in phenomenology, course, correlates and treatment response [3, 25]. This distinction between RAD and DSED is well founded in young children [3, 25, 27] and is evident in school-aged children [11, 28]. Although less studied in adolescence, confirmatory factor analysis (CFA) has revealed a clear distinction between RAD and DSED in foster care adolescents [8]. Therefore, we hypothesize that symptoms of RAD and DSED in adolescents living in residential youth care (RYC) will form two definite clusters, assessed both with a dimensional factor analytic approach and a categorical/typological latent profile approach. If so, the discriminant validity of RAD and DSED vis-à-vis other psychiatric disorders will be studied.

In summary, by approaching all adolescents living in Norwegian RYC and using in-depth semi-structured psychiatric interviews, we (1) estimate the prevalence of symptoms and the diagnosis of RAD and DSED in a high-risk group of adolescents mainly unexposed to early institutionalization; (2) hypothesize that RAD and DSED symptoms in adolescence cluster corresponding to the DSM-5 definitions using both CFA and latent profile analysis (LPA); and (3) hypothesize that RAD and DSED symptoms in adolescence tap into diagnostic phenomena that are distinct from other psychiatric disorders.

## Methods

### Participants

All residents aged 12–23 years living in Norwegian RYC between 2011 and 2014 were invited to participate in the research project Mental Health in Adolescents living in Residential Youth Care [29]. A flowchart of the recruitment process is depicted in Figure S1 (Supplementary material). In short, 400 of 601 eligible adolescents consented to participate. Participants with a completed primary contact interview (*N* = 381) yielding information about RAD and DSED had the following characteristics: 57.7% girls (*n* = 220); age 12.2–20.2 years (*M* = 16.7, SD = 1.4); age at first out-of-home placement: *M* = 12.5 years (SD = 3.9), median 14.0 years (details in Table S1); mean number of out-of-home placements: 3.3 (SD = 2.4); and 78% ethnic Norwegian.

### Setting

The Norwegian child protection services (CPS) provide support to approximately 2.5% (*n* = 37,124 ultimo 2014, which was the final year of data collection in the present study) of individuals aged 0–22 years in Norway, primarily aiming to aid children within their families [30]. Placement in out-of-home care (0.99%, *n* = 14,495 in 2014) is only relevant when in-family support fails to fulfil a child’s basic needs [30]. In that case, foster care is both preferred and most common; only a small minority (0.09%, *n* = 1255 in 2014) of the population aged 0–22 years are in RYC [30], representing the last resort in Norwegian CPS [31]. In accordance with the CPS criteria [32], looked-after minors have likely been exposed to social neglect, inadequate care or maltreatment prior to out-of-home placement. A previous study, also part of the current mental health project on adolescents in Norwegian RYC, revealed that 78.1% of girls and 60.4% of boys self-reported exposure to maltreatment, and the authors concluded that virtually all had likely experienced some form of neglect [33]. Moreover, adolescents in the current mental health project have reported high levels of parental chronic illness, mental illness or drug use [29], indicating increased risk of pathogenic care prior to out-of-home placement [10]. Furthermore, serious neglect was reported for 86% of school-aged foster children in Norway [34], a group regarded as exposed to less risk than adolescents in RYC. Therefore, participants were considered to likely fulfil the RAD/DSED criteria of exposure to insufficient care.

RYC institutions in Norway are typically small units resembling family homes and are strictly regulated by law, with explicit requirements regarding quality and internal control [35]. RYC institutions are obliged to ensure ethically and professionally sound methods based on currently accepted knowledge. There are defined material requirements regarding maintenance, hygiene, adequate play areas and other social and creative activities. Staff requirements specify that the personnel must have adequate levels of professional competency and that the worktime arrangement must ensure continuity and stability for the adolescents living in RYC. There are specific competency requirements for the leaders of RYCs and for the professional training and guidance of all staff. Every child has a designated primary contact among the staff who on a daily basis has individual responsibility for that particular child and who aims to fulfil the various roles of a primary caregiver. Because of the small size of Norwegian RYC institutions, which typically house three to five residents, and the focus on the continuity and stability of personnel, the setting generally allows the primary contacts to know the residents well and vice versa. As a rule, each resident gets to keep their designated primary contact throughout their stay, and 90% of the participants reported having lived in RYC for a minimum of 3 months prior to the data collection [36]. As a whole, primary contacts were therefore considered to have known the residents long enough to enable the establishment of a trusting relationship and to have acquired sufficient knowledge of the residents to be reliable informants.

### Procedure

Data were collected at the RYC institutions by four trained research assistants with relevant higher education and extensive work experience with children and families. Semi-structured psychiatric interviews were completed with the adolescents and their primary contacts. Data were collected from June 2011 until July 2014. The Norwegian Regional Committee for Medical and Health Research Ethics, Central Norway approved the study. Written, informed consent was acquired for all participants.

### Instruments

#### Interview with the adolescents

The Child and Adolescent Psychiatric Assessment (CAPA) [37] is a semi-structured psychiatric interview for children and adolescents designed to determine a wide range of DSM-IV-defined psychiatric disorders. The CAPA includes both required and optional follow-up questions and information concerning the impairment, intensity, onset, duration and frequency of symptoms. Interviewers must ensure that interviewees understand the questions and probe until clarifying whether symptoms are present or not, according to predefined criteria. In this study, symptoms of the following diagnostic categories were assessed using CAPA: major depressive disorder (MDD); dysthymia; generalized anxiety disorder (GAD); panic attacks; other anxiety disorders; conduct disorder (CD); oppositional defiant disorder (ODD); and posttraumatic stress disorder (PTSD). Details are in Table S2.

#### Interview with the adolescents’ primary contacts

Previous research indicates that adolescents are less reliable informants regarding symptoms of attention deficit hyperactive disorder (ADHD) and autism spectrum disorder (ASD) than adults who know them well [38–40]. Likewise, as self-acknowledging signs of RAD and DSED would require substantial mentalisation and individuals with these disorders likely have lacked the supportive caregiving relationships necessary to promote this ability [41], the adolescents were expected to be sub-optimal informants of RAD and DSED symptoms. Therefore, symptoms of ADHD, ASD, RAD and DSED were assessed using the adolescents’ primary contacts as informers. The caregiver version of CAPA was used to assess ADHD, whereas RAD and DSED were assessed using the RAD module in the Preschool Age Psychiatric Assessment (PAPA) [24]. As ASD symptoms are not included in CAPA, ASD was evaluated using the Asperger Syndrome Diagnostic Interview (ASDI) [42]. Details are in Table S2.

Regular and random checks of the interviews were conducted to ensure adherence to the interview protocol and to prevent interviewer drift. A randomly drawn proportion (*n* = 42; 10.5%) of interview audio recordings were re-coded by blinded raters to provide inter-rater reliability estimates. In previous findings, inter-rater reliability for DSM-IV diagnoses estimated by Gwet’s AC_1_ and rater pair agreement rate (%) ranged from AC_1_ = 0.74 to AC_1_ = 1.0 (83–100%) [29].

#### Assessment of RAD and DSED

The PAPA is a caregiver report preschool version of CAPA, including a module with questions targeted at assessing DSM-IV inhibited and disinhibited RAD, corresponding to RAD and DSED as defined in DSM-5. Table 1 shows the 15 available items, the corresponding DSM-5 criteria for RAD and DSED and the frequency of symptoms that satisfied predefined severity levels for diagnostic contribution, requiring high rates of symptom load and functional impairment (specifications in Table 1, notes). In the computation of prevalence rates, adolescents who fulfilled the RAD A1 criteria (minimally seeks comfort) and a minimum of one item for two or more B criteria (minimal social and emotional responsiveness; limited positive affect; unexplained irritability/sadness/fearfulness during non-threatening interactions) were classified as having RAD. We lacked a measure of the RAD A2 criteria ‘rarely or minimally responds to comfort when distressed’ [1] and were therefore unable to include this in the diagnostics. In accordance with DSM-5, individuals with co-occurring RAD and ASD (*n* = 5) were considered not to have RAD when calculating disorder prevalence rates. Adolescents who fulfilled at least two of the DSED criteria were classified as having DSED.

**Table 1:** Symptom frequencies and categorization according to DSM-5 criteria for reactive attachment disorder (RAD) and disinhibited social engagement disorder (DSED)

RAD criteria	RAD items	*n*	%
A1 Minimally seeks comfort	Does not seek comfort when distressed^a^	62/377	16.4
B1 Minimal social and emotional responsiveness	Inhibited social interactions	69/381	18.1
	Lacks interest in people^b^	26/376	6.9
	Avoids eye contact	39/380	10.3
	Avoids physical contact^c^	81/380	21.3
	Lacks emotional sensitivity	80/375	21.3
B2 Limited positive affect	Difficulty being affectionate	100/377	26.5
	Constricted facial expression	71/375	18.9
B3 Unexplained irritability/sadness/fearfulness	Highly ambivalent and contradictory responses^d^	128/370	34.6
	Negative reunion responses^e^	9/376	2.4
	Hypervigilance^f^	31/361	8.6

DSED criteria	DSED items	*n*	%
A1 Reduced reticence with unfamiliar adults	Indiscriminate adult relationships^g^	28/380	7.4
A2 Overly familiar verbal or physical behaviour	Indiscriminate peer relationships^g^	39/374	10.4
A3 Diminished checking back with caregiver	Minimal checking with caregiver in unfamiliar settings^g^	13/368	3.5
A4 Willingness to go off with unfamiliar adult	Indiscriminate willingness to leave with unfamiliar adult^g^	41/371	11.1

^a^Regularly, in most activities

^b^Adult family members and peers

^c^Often or always

^d^Affecting ≥ 2 activities and interfering with relationships

^e^Positive interaction cannot be restored within one hour

^f^Interfering with ≥ 2 activities

^g^To a problematic degree

### Statistical analysis

Because PAPA has been constructed and validated for use in young children and studies differentiating RAD and DSED in adolescence are scarce, we used two complimentary approaches to evaluate whether the PAPA measure defines and distinguishes RAD and DSED in adolescence. First, conceptualizing RAD/DSED as latent dimensional factors, one- versus two-factor CFA solutions were compared, applying commonly used fit indices. A two-step procedure for chi-square difference testing using the diff-test option was performed, as advised when using a means- and variance-adjusted weighted least squares (WLSMV) estimator [43]. Second, conceptualizing RAD/DSED as categorical constructs, LPA was used to compare 1–4 classes according to their entropy, prevalence, and the Vuong-Lo-Mendell-Rubin (VLMR) likelihood ratio test of *k* versus *k* − 1 classes [44].

To determine discriminant validity, one-factor solution CFAs containing symptoms of RAD/DSED and a differential disorder (in separate models) (i.e. MDD, dysthymia, GAD, panic attacks, other anxiety disorders, ADHD inattentive type, ADHD hyperactive/impulsive type, CD, ODD, PTSD and ASD) were compared to two-factor solutions (as above, using model fit indices and chi-square difference testing), where symptoms loaded separately on RAD/DSED and each differential disorder. Due to low prevalence rates in the sample [29], the following psychiatric disorders were not analysed: bipolar disorder, obsessive compulsive disorder, bulimia, anorexia nervosa and Tourette syndrome. Although the diagnosis of PTSD was also rare [29], symptoms of PTSD were sufficiently frequent (range *n* = 6–64) for factor analysis [45], and were therefore included.

The LPA and all CFAs were conducted in Mplus, version 8 [43]; symptoms were treated as categorical variables using a WLSMV estimator. All other analyses were conducted in SPSS version 25.0 [46].

## Results

### Prevalence

As shown in Table 1, the frequency of individual RAD symptoms ranged from 2.4 to 34.6%; negative reunion responses were infrequent, whereas difficulties being affectionate and highly ambivalent and contradictory responses were prevalent. The frequency of DSED symptoms ranged from 3.5 to 11.1%; minimal checking with caregivers in unfamiliar settings was relatively rare, whereas indiscriminate willingness to leave with an unfamiliar adult and indiscriminate behaviour with peers were more frequent. In all, 16.3% (95% CI 12.6–20.0%; *n* = 62) fulfilled the criteria for either RAD or DSED, with 8.7% having RAD (95% CI 6.0–11.0%; *n* = 33), 8.1% having DSED (95%CI 5.4–10.9%; *n* = 31) and 0.5% (*n* = 2) having both disorders. There were no gender differences for RAD (57.6% girls; age adjusted OR = 1.06, 95% CI 0.51–2.21, *p* = 0.88), but girls were overrepresented among adolescents with DSED (80.6% girls; age adjusted OR = 3.90, 95% CI 1.52–10.01, *p* = 0.005). No differences were found between adolescents with and without RAD or DSED for the remaining characteristics (age, age at first placement, number of out-of-home placements and ethnicity).

### RAD versus DSED

In the CFA, a two-factor solution, with RAD and DSED as separate latent variables as in DSM-5, fit the data better than a one-factor solution, corresponding to the DSM-IV diagnostic construct of a single RAD disorder (Table 2). The comparative fit index and the Tucker–Lewis index showed suboptimal values in the two-factor model of RAD and DSED. However, the root mean square error of approximation was satisfactory, having a narrow confidence interval with an upper limit of < 0.06, signalling that the hypothesized two-factor model of RAD and DSED fit the data well enough [47]. Further, the correlation between the two factors was modest (Figure S2; Est. = 0.23, *p* = 0.010). Although a k2 LPA fit the data better than a one-class solution, VLMN-2LL = 311.42, *p* < 0.001, a k3 solution proved better than a k2 solution, VLMN -2LL = 100.64, *p* < 0.001, whereas a k4 solution, VLMN -2LL = 49.39, *p* = 0.24, did not improve fit. Entropy for the k3 was 0.79, with estimated prevalence rates of 32.0% (‘RAD’), 12.5% (‘DSED’) and 55.5% (‘Neither’). Therefore, the three-class solution was preferred. The RAD and DSED classes were characterized by symptoms of RAD and DSED, respectively (Table 3), except for the RAD symptoms ‘lacks emotional sensitivity’ and ‘highly ambivalent and contradictory responses’, also being found in the DSED class. In addition, ‘negative reunion responses’ and ‘hypervigilance’ were found equally frequently in the RAD and DSED classes.

**Table 2:** Confirmatory factor analysis: symptoms of reactive attachment disorder (RAD), disinhibited social engagement disorder (DSED) and differential psychiatric disorders. Model fit indices for one-factor models (1) and two-factor models (2.)

Disorders		Model	Chi-square				RMSEA	90% CI	CFI	TLI	Diff. test		
			χ^2^	*df*	*p*	χ^2^/*df*					χ^2^	*df*	*p*
RAD vs. DSED		1	227.60	90	< 0.01	2.5	0.06	0.05–0.07	0.76	0.71	50.4	1	< 0.001
		2	151.17	89	< 0.01	1.7	0.04	0.03–0.05	0.89	0.87			
MDD	RAD	1	574.83	170	< 0.01	3.4	0.08	0.07–0.09	0.68	0.65	88.72	1	< 0.001
		2	245.22	169	< 0.01	1.5	0.03	0.02–0.04	0.94	0.93			
	DSED	1	175.33	65	< 0.01	2.7	0.07	0.06–0.08	0.90	0.88	40.84	1	< 0.001
		2	91.44	64	0.01	1.4	0.03	0.02–0.05	0.98	0.97			
Dysthymia	RAD	1	478.79	135	< 0.01	3.6	0.08	0.07–0.09	0.63	0.58	81.41	1	< 0.001
		2	173.96	134	0.01	1.3	0.03	0.01–0.04	0.96	0.95			
	DSED	1	107.08	44	< 0.01	2.4	0.06	0.05–0.08	0.91	0.89	31.31	1	< 0.001
		2	39.84	43	0.61	0.9	0.00	0.00–0.03	1	1.01			
GAD	RAD	1	504.60	152	< 0.01	3.3	0.09	0.08–0.09	0.93	0.92	82.25	1	< 0.001
		2	235.98	151	< 0.01	1.6	0.04	0.03–0.05	0.98	0.98			
	DSED	1	151.74	54	< 0.01	2.8	0.08	0.06–0.09	0.98	0.98	47.12	1	< 0.001
		2	70.63	53	0.05	1.3	0.03	0.00–0.05	1	1.00			
Panic attack	RAD	1	614.10	230	< 0.01	2.7	0.07	0.07–0.08	0.98	0.98	82.84	1	< 0.001
		2	255.74	229	0.11	1.1	0.02	0.00–0.03	1	1.00			
	DSED	1	174.05	104	< 0.01	1.7	0.05	0.03–0.06	1	1.00	49.17	1	< 0.001
		2	97.69	103	0.63	1.0	0.00	0.00–0.03	1	1.00			
PTSD	RAD	1	691.58	405	< 0.01	1.7	0.05	0.04–0.05	0.96	0.96	89.11	1	< 0.001
		2	444.88	404	0.08	1.1	0.02	0.00–0.03	1.0	1.0			
	DSED	1	285.53	230	0.01	1.2	0.03	0.02–0.04	0.99	0.99	42.07	1	< 0.001
		2	246.41	229	0.20	1.1	0.01	0.00–0.03	1.0	1.0			
Other anxiety	RAD	1	499.48	209	< 0.01	2.4	0.07	0.06–0.07	0.71	0.68	75.67	1	< 0.001
		2	262.55	208	0.01	1.3	0.03	0.02–0.04	0.95	0.94			
	DSED	1	176.85	90	< 0.01	2.0	0.06	0.04–0.07	0.90	0.88	41.12	1	< 0.001
		2	123.92	89	0.01	1.4	0.04	0.02–0.05	0.96	0.95			
ADHD-1	RAD	1	552.98	170	< 0.01	3.3	0.08	0.07–0.08	0.80	0.77	63.66	1	< 0.001
		2	271.01	169	< 0.01	1.6	0.04	0.03–0.05	0.95	0.94			
	DSED	1	163.72	65	< 0.01	2.5	0.06	0.05–0.08	0.94	0.93	36.79	1	< 0.001
		2	94.24	64	0.01	1.5	0.04	0.02–0.05	0.98	0.98			
ADHD-2	RAD	1	689.53	170	< 0.01	4.1	0.09	0.08–0.10	0.77	0.75	80.70	1	< 0.001
		2	400.45	169	< 0.01	2.4	0.06	0.05–0.07	0.90	0.89			
	DSED	1	236.38	65	< 0.01	3.6	0.08	0.07–0.10	0.92	0.90	40.76	1	< 0.001
		2	174.24	64	< 0.01	2.7	0.07	0.06–0.08	0.95	0.93			
CD	RAD	1	415.03	209	< 0.01	2.0	0.05	0.04–0.06	0.65	0.62	78.78	1	< 0.001
		2	247.96	208	0.03	1.9	0.02	0.01–0.03	0.93	0.93			
	DSED	1	171.73	90	< 0.01	1.9	0.05	0.04–0.06	0.69	0.64	48.56	1	< 0.001
		2	100.21	89	0.20	1.1	0.02	0.00–0.03	0.96	0.95			
ODD	RAD	1	405.35	152	< 0.01	2.7	0.07	0.06–0.07	0.60	0.55	86.67	1	< 0.001
		2	206.36	151	< 0.01	1.4	0.03	0.02–0.04	0.91	0.90			
	DSED	1	102.54	54	< 0.01	1.9	0.05	0.03–0.06	0.87	0.84	24.65	1	< 0.001
		2	63.55	53	0.15	1.2	0.02	0.00–0.04	0.97	0.96			
ASD	RAD	1	253.14	119	< 0.01	2.1	0.05	0.04–0.06	0.92	0.90	15.20	1	< 0.001
		2	219.44	118	< 0.01	1.9	0.05	0.04–0.06	0.93	0.92			
	DSED	1	144.77	35	< 0.01	4.1	0.09	0.08–0.11	0.79	0.73	41.43	1	< 0.001
		2	67.58	34	< 0.01	2.0	0.05	0.03–0.07	0.94	0.91			

*ADHD-1* attention deficit hyperactive disorder (ADHD) attention deficit type, *ADHD-2* ADHD hyperactive and impulsive type; *ASD* autism spectrum disorder, *CD* conduct disorder, *CFI* comparative fit index, *CI* confidence interval, *Diff. test* Chi-square two-step difference testing of Model 1 versus Model 2 using the Mplus diff-test option, *GAD* generalized anxiety disorder, *MDD* major depressive disorder, *ODD* oppositional defiant disorder, *PTSD* posttraumatic stress disorder, *RMSEA* root mean square error of approximation, *TLI* Tucker–Lewis index

**Table 3:** Percentage with symptoms of reactive attachment disorder (RAD) and disinhibited social engagement disorder (DSED) in 3 latent classes. Latent profile analysis

Item	Criteria	Class 1 (32.0%) RAD	Class 2 (12.5%) DSED	Class 3 (55.5%) Neither
y1	Inhibited social interactions	41.9	9.0	6.4
y2	Lacks interest in people	18.3	0.0	2.0
y3	Does not seek comfort when distressed	34.1	0.0	10.1
y4	Lacks emotional sensitivity	42.6	29.6	7.3
y5	Difficulty being affectionate	68.9	5.6	7.1
y6	Avoids physical contact	51.9	19.3	4.2
y7	Constricted facial expression	48.5	10.8	4.0
y8	Avoids eye contact	28.3	0.0	2.1
y9	Highly ambivalent and contradictory responses	55.2	44.5	20.3
y10	Negative reunion responses	5.1	4.4	0.4
y11	Hypervigilance	14.3	14.5	4.1
y12	Indiscriminate adult relationships	6.2	32.8	2.3
y13	Indiscriminate peer relationships	4.8	60.9	2.8
y14	Indiscriminate willingness to leave with unfamiliar adult	10.3	61.4	0.3
y15	Minimal checking with caregiver in unfamiliar settings	5.2	14.6	0.0

### Discriminant validity

Because a distinction between RAD and DSED was supported by the above, subsequent analyses were conducted separately for the two disorders. For all differential disorders, the examined two-factor solutions distinguishing between symptoms of RAD or DSED and the disorder in question evidenced better fit than one-factor combined RAD or DSED and differential psychiatric disorder solutions (Table 2). Even though a two-factor solution of RAD and ASD proved to have better fit than a one-factor solution, it should be observed that the two latent constructs correlated highly (Table 4). In contrast, the correlations between RAD and other disorders were modest (Table 4). Between DSED and other disorders, the correlations were modest for MDD, GAD, panic attack, PTSD, other anxieties, CD and ASD and were moderate for dysthymia, ADHD (both types) and ODD (Table 4).

**Table 4:** Covariance of symptoms of reactive attachment disorder (RAD) and disinhibited social engagement disorder (DSED) with symptoms of other psychiatric disorders in adolescence

Disorder	RAD			DSED		
	Estimate	S.E	*p*	Estimate	S.E	*p*
MDD	0.24	0.08	0.003	0.36	0.09	< 0.001
Dysthymia	0.22	0.09	0.012	0.42	0.10	< 0.001
GAD	0.32	0.07	< 0.001	0.24	0.10	0.015
Panic attack	0.21	0.09	0.019	0.22	0.13	0.094
PTSD	0.23	0.09	0.012	0.30	0.10	0.004
Other anxiety	0.23	0.08	0.003	0.20	0.10	0.040
CD	− 0.12	0.09	0.19	0.11	0.12	0.38
ODD	0.11	0.10	0.27	0.45	0.11	< 0.001
ADHD-1	0.38	0.07	< 0.001	0.45	0.08	< 0.001
ADHD-2	0.27	0.06	< 0.001	0.44	0.08	< 0.001
ASD	0.84	0.04	< 0.001	0.28	0.10	0.004

*S.E*. standard error, *p* two-tailed *p* value, *ADHD-1* attention deficit hyperactive disorder (ADHD) attention deficit type, *ADHD-2* ADHD hyperactive and impulsive type, *ASD* autism spectrum disorder, *CD* conduct disorder, *GAD* general anxiety disorder, *MDD* major depressive disorder, *ODD* oppositional defiant disorder, *PTSD* posttraumatic stress disorder

## Discussion

To help clarify existing controversy concerning RAD and DSED as diagnostic constructs in adolescence, we studied their construct validity, including structural and discriminant validity, in a high-risk group of adolescents living in Norwegian RYC. An interviewer-based measure developed and validated for young children revealed frequencies of RAD symptoms ranging from 2 to 35% and of DSED symptoms ranging from 4 to 11%. The prevalence according to DSM-5 criteria was 9% RAD and 8% DSED, with 0.5% having both disorders. Furthermore, dimensional (CFA) and categorical/typological (LPA) approaches converged in discriminating between RAD and DSED. Finally, both RAD and DSED were distinguishable from MDD, dysthymia, various anxiety disorders, PTSD, ADHD, CD, ODD and ASD. Taken together, the results suggest that in adolescence, RAD and DSED are distinct and valid diagnostic constructs not accounted for by more common psychopathology.

### Prevalence

Although all measured symptoms of RAD and DSED were present, negative reunion responses and to some extent minimal checking were rather infrequent, perhaps indicating that these behaviours are not age-typical symptoms of RAD and DSED, respectively, in adolescence. Some RAD symptoms were more prevalent than any DSED symptom. This might be due to the inclusion of almost three times as many RAD as DSED symptoms, thus increasing the probability that some RAD symptoms would be prevalent. Such a view is consonant with the fact that many participants reported RAD symptoms without qualifying for a RAD disorder, whereas the frequencies of the most prevalent DSED symptoms were more consonant with the prevalence of DSED disorder. Accordingly, the PAPA RAD items may look less specific than the DSED items in adolescence. However, the factor analyses indicated that the RAD items were quite specific for RAD.

The prevalence of RAD and DSED vary greatly depending on risk exposure, thereby limiting generalizability. Nonetheless, our findings (16% RAD/DSED) are concordant with the prevalence (19% inhibited/disinhibited RAD, DSM-IV) among school-aged foster children in Norway [34], a group with lower risk of exposure than adolescents living in Norwegian RYC. Compared to the current RYC sample, the foster children had significantly lower mean age at first out-of-home placement (3.74 years (SD = 2.98) versus 12.5 years (SD = 3.9)), lower mean number of placements (0.90 (SD = 0.85) versus 3.3 (SD = 2.4)) and lower point prevalence of psychiatric disorders (50.9% versus 76.2%) [34]. In a systematic review that included 92 studies, higher age at first out-of-home placement and a higher number of placements were identified as key factors associated with a range of negative health-related outcomes [48]. The Norwegian CPS has a family-preserving focus, typically providing in-home interventions for three years prior to the first out-of-home placement of a child [49]. In result, children may experience prolonged exposure to pathogenic care if living with parents who despite interventions by CPS prove unable to provide their child with the necessary nurture and developmental support [50]. These considerations taken together, adolescents in Norwegian RYC may therefore be considered at increased risk of early adversity compared to foster children in Norway, and the comparability of prevalence rates for these two groups indicates the unlikelihood of RAD/DSED being over-diagnosed in this study. The preponderance of girls with DSED was surprising, as it is undescribed in previous research in adolescence [6, 8, 9]. Possible explanations include rater bias, such as primary contacts being more concerned by indiscriminate behaviour in girls than boys, or sample bias, such as gender differences in types and frequencies of adverse experiences [33].

### RAD versus DSED

Finding a two-factor structure and two corresponding clusters of RAD and DSED in adolescence is consistent with the understanding of RAD/DSED in younger children (reviewed in [2]; [25]) and is concordant with the revision into two distinct disorders in DSM-5. Even so, the RAD symptoms of ambivalence and lack of emotional sensitivity were also seen in the DSED profile, and hypervigilance and negative reunion responses were equally frequent in both profiles. This is consonant with the high correlation found between symptoms of DSED and the RAD B criteria in foster-placed adolescents [8] and may reflect that such potential effects of relational trauma and inadequate developmental support are not specific to RAD but may also co-exist with DSED behaviour in adolescence. Replications are needed (also in young children) before considering possible implications of the RAD/DSED criteria.

### Discriminant validity

In line with the understanding of RAD and DSED in younger children [11–15] is the finding that symptoms of RAD/DSED are distinct from symptoms of other psychiatric disorders in adolescence. Due to overlapping social difficulties, a significant clinical challenge is differentiating RAD from ASD. Finding a high covariance between RAD and ASD is reflective of this. Nonetheless, the model differential test indicated that RAD and ASD are best conceptualized as different disorders, supporting previous findings that RAD and ASD are indeed differentiable [12, 51].

### Strengths and limitations

The use of in-depth psychiatric interviews for diagnostic assessment and examining a nation-wide very high-risk population are clear strengths. Given the lack of validated assessment tools for RAD and DSED in adolescents, we used a DSM-IV-based diagnostic caregiver interview developed and validated for young children. Although this could be questioned, it is arguably a methodological strength because one of the key questions is whether RAD and DSED, as seen in young children and defined by DSM, also exist in adolescents. After the completion of data collection in the current study, an instrument – the RAD and DSED assessment interview (RADA) [8] was developed by a different research group specifically to assess DSM-5-defined RAD and DSED in adolescents. Critically, there is substantial overlap between RADA and the measurement of RAD/DSED as per PAPA, lending support to the age relevance of the items used in the current study. Therefore, we expect that a potential adjustment of PAPA to DSM-5 and adolescent age would alter or add very few items and not critically affect the factor structure we revealed. However, to assess possible heterotypic continuity would require longitudinal studies, and to assess whether the diagnostic phenomena we describe differ importantly from early institutionalized samples would require comparative studies.

Further, possibly important limitations are the lack of observational data and multiple methods of assessment (triangulation), contrary to expert recommendations for the clinical assessment of RAD and DSED [2, 52]. Although common in research on RAD and DSED, also in adolescent samples [6–8], the sole use of caregiver report could generate rater bias. In a school-aged sample, a study of the convergence between another semi-structured interview (disturbance of attachment interview, DAI) with primary caretakers and the clinical diagnosis of RAD and DSED using DSM-5 criteria (based on clinical observation and the child’s attachment history) found 33% of the children to be categorized with RAD or DSED based on the DAI, whereas only 18% received a clinical RAD or DSED diagnosis. The DAI was found to be consistent with a clinical diagnosis of RAD or DSED in 75% of the cases and was categorized as having only fairly strong predictive validity for RAD and DSED [53]. Notably, the DAI diagnoses only required three positive RAD items or two positive DSED items, respectively, where items were positively scored if either somewhat/sometimes or considerably/frequently present. In the clinical diagnostics, however, the DSM-5 criteria were applied, setting stricter requirements for the fulfilment of RAD and DSED. The authors conclude that diagnosing RAD and DSED based solely on a semi-structured interview (DAI) with primary caretakers may lead to overdiagnosis. Therefore, as we lacked direct observational measures of attachment behaviours, we took action to reduce the risk of overdiagnosis in this study. First, we predefined requirements with high levels of symptom load and functional impairment for positive scores (e.g. symptoms must affect at least two activities, interfere with relationships or be present to a problematic degree). Second, we organized the RAD and DSED items according to the DSM-5 A and B criteria for RAD and the A1–A4 criteria for DSED, assuring that the diagnostic algorithms were met. Although we cannot exclude the possibility of rater bias, the diagnostic procedure used herein is clearly stricter than the DAI-based diagnostic mentioned above. Therefore, we expect the risk of false positive RAD and DSED diagnoses to be more limited. Further, numerous studies, albeit in younger children, have shown considerable convergence between observational data and caregiver reports for symptoms of RAD/DSED (reviewed in [3]), also using PAPA [15], thus lending support to the validity of our findings.

Another possible limitation regarding the assessment of RAD (but not DSED) in adolescents living in RYC is that absent or aberrant attachment behaviour toward their primary contact in RYC may not be representative of attachment behaviours toward previous caregivers. Further, as we only have general knowledge of the Norwegian laws, regulations and practices for out-of-home placements, we can substantiate but not be fully certain that all participants identified as having RAD or DSED satisfy the diagnostic criteria of exposure to extremes of insufficient care and symptom debut before age 5 (RAD only). Additionally, the DSM-5 RAD criteria require both minimal seeking and responding to comfort, whereas we lacked information on the latter, potentially inflating the reported prevalence of RAD. Nonetheless, we consider overdiagnosis of RAD/DSED to be unlikely, given the general high-risk nature of the sample and prevalence comparability to school-aged foster children in Norway.

The use of different informants (primary contact and adolescents) for different types of psychopathology may be regarded as a strength, as it allows avoiding common-rater bias in many of our findings. On the other hand, the use of different informants may be problematic in assessing discriminant validity, as differences may at least partially be due to informant discrepancies. However, symptoms of ADHD and ASD, which like RAD and DSED were caregiver-informed, were also found to be distinct from those of RAD and DSED. As differentiation of DSED from ADHD [1] and RAD from ASD [1, 12] is considered to be particularly challenging, this lends support to our overall findings of discriminant validity. Although our results demonstrate that RAD and DSED are distinct from many psychiatric disorders, this conclusion is limited to the disorders studied.

### Clinical implications

Undoubtedly, identifying RAD and DSED while overlooking other common disorders may be detrimental due to missed treatment of other treatable disorders [54]. Yet, overlooking RAD or DSED may be equally damaging, as an incomplete or incorrect case formulation may reduce the likelihood of adequate developmental support for a child. In cases with RAD or DSED, caregivers may need specialized interventions, aiming to enhance their sensitivity, emotional availability and commitment to the child [2, 3]. Such interventions are not necessarily offered when treating adolescents who have other psychiatric disorders without RAD or DSED (e.g. depression, anxiety, PTSD, ADHD, ASD). Inadequate caregiver support may not only lead to continued suffering but also the increased risk of placement breakdown [55], further adding to the individual burdens and societal costs of RAD and DSED. By contrast, correctly identifying and acknowledging RAD and DSED in adolescence may enhance the likelihood of meeting the child’s developmental needs. Based on our results indicating the existence and validity of these disorders in adolescence, we advocate that clinicians assess and acknowledge RAD and DSED beyond early childhood. Moreover, the relatively high prevalence rates of RAD and DSED among adolescents in RYC warrant that all RYC personnel receive appropriate training and education (ensuring knowledge of treatment recommendations [2]) to enable them to understand the underlying reasons for the residents’ behaviours and to provide developmentally supportive relational experiences. As for other primary caretakers, the RYC personnel may need specialized guidance to help them enhance and maintain their sensitivity and emotional availability when interacting with the residents over time, as RAD and DSED behaviour may be relationally challenging and can easily provoke negative responses from the caretakers, further aggravating rather than ameliorating the underlying struggles of the child.

The high covariance between RAD and ASD and the risk of misinterpreting, for example, RAD as depression or anxiety (or vice versa) or DSED as ADHD (or vice versa) warrants clinical thoroughness and comprehensive psychiatric assessment of individuals exposed to childhood adversity, as advised in the practice parameter for RAD and DSED [2]. Further, high-risk groups, such as individuals living in RYC, should have easy access to high-quality psychiatric assessment and care.

Because RAD and DSED are in principle preventable, as their common aetiology involves exposure to extremes of insufficient care, measures ensuring adequate care and support for all young children and their families could have long-lasting benefits for the individuals, families and societies involved, empowered by further collaboration between researchers, child protection services, clinicians, public health planners and policy makers. Further research on associations between RAD/DSED and other mental health factors, as well as homotypic and heterotypic continuity into adulthood, could illuminate possible treatment targets in adolescence.

## Conclusion

In a very high-risk RYC adolescent sample, RAD and DSED emerged as two distinct latent factors not accounted for by other common psychiatric disorders. RAD and DSED are not uncommon among adolescents in RYC. To alleviate individual suffering and societal costs and because RAD and DSED are preventable and may imply treatment approaches not otherwise offered, it is pertinent that RAD and DSED in both childhood and adolescence be acknowledged by clinicians, child protection services, public health planners and policy makers.

## Electronic supplementary material

Below is the link to the electronic supplementary material.

Supplementary file1 (DOCX 176 kb)
