# Supplementary material for: Validity of reactive attachment disorder and disinhibited social engagement disorder in adolescence
Source: Eur Child Adolesc Psychiatry. 2019 Dec 12;29(10):1465–76. doi: 10.1007/s00787-019-01456-9 (PMC7501108; doi:10.1007/s00787-019-01456-9)
Supplement: Supplementary file 1 — Supplementary file1 (DOCX 176 kb) [file 787_2019_1456_MOESM1_ESM.docx]

# Supplementary Material (Online Resource 1)

**Article:** Validity of Reactive Attachment Disorder and Disinhibited Social Engagement Disorder in Adolescence

**Journal:** European Child and Adolescent Psychiatry

**Authors:** Astrid R. Seim, Thomas Jozefiak, Lars Wichstrøm and Nanna S. Kayed

**Corresponding author:** Astrid Røsland Seim, e-mail: astrid.r.seim@ntnu.no, Department of Mental Health, Faculty of Medicine and Health Sciences, NTNU Norwegian University of Science and Technology, Trondheim, Norway

Figure S1. Participants and recruitment. *RYC* residential youth care; *PCI* primary contact interview; *CAPA* Child and Adolescent Psychiatric Assessment.
^a^ Exclusion criteria: Adolescents in acute placement and unaccompanied minors without asylum in Norway were excluded from the study for ethical reasons, as they were considered to be in such a high state of crisis that data collection should not be prioritized. Those with insufficient Norwegian language qualifications to be interviewed were also excluded. Nonetheless, for adolescents registered as unaccompanied minors living in regular RYC who had received a residence permit from Norwegian authorities and who were sufficiently fluent in the Norwegian language, requesting permission to participate in the study was considered ethically acceptable.
^b^ To evaluate the representativeness of the data for participants with available CAPA and PCI data (*n*=322), mean scores of the Child Behavior Checklist (CBCL) syndrome subscales were compared to participants where CAPA was not completed (*n*=59). None of the subscales showed statistically significant differences in mean values. Therefore, the participants with both CAPA and PCI were considered to be representative of the whole sample when comparing RAD and DSED symptoms (from PCI) to symptoms of other disorders (from CAPA).

Table S1. Distribution of age at first out-of-home placement

| Age in years | *n* | Cumulative % |
| --- | --- | --- |
| 0 | 2 | .5 |
| 1 | 7 | 2.3 |
| 2 | 9 | 4.7 |
| 3 | 5 | 6.0 |
| 4 | 3 | 6.8 |
| 5 | 7 | 8.6 |
| 6–11 | 62 | 24.9 |
| 12–17 | 281 | 98.7 |
| Missing | 5 | 100 |
| Total | 381 | 100 |

Table S2. Symptom clusters for differential psychiatric disorders included in the confirmatory factor analysis (CFA)

| Disorder | CAPA-derived symptoms included in CFA |
| --- | --- |
| MDD | Depressed or irritable mood  Anhedonia or loss of interest  Weight loss/gain or appetite disturbance  Insomnia or hypersomnia  Psychomotor agitation/retardation  Fatigue or loss of energy  Feelings of worthlessness or guilt  Problems with thinking or deciding  Suicidal thoughts, suicidal plan or attempted suicide |
| Dysthymia | Prolonged depressed mood (>45 days)  Weight loss or gain  Insomnia or hypersomnia  Loss of energy  Low self-esteem  Problems with thinking  Hopelessness |
| GAD | Nervous tension  Anxious foreboding  Feeling keyed up or on edge  Being easily fatigued  Difficulty concentrating or mind going blank  Irritability  Muscle tension  Sleep disturbance |
| Panic attack^a^ | Discrete period of intense fear or discomfort  Palpitations or accelerated heart rate  Sweating  Trembling or shaking  Sensations of shortness of breath or smothering  Feeling of choking  Chest pain or discomfort  Nausea or abdominal distress  Feeling dizzy or lightheaded  Depersonalization  Fear of going crazy  Fear of dying |
| PTSD | Traumatic life event  Acute emotional response to traumatic life event  Distressing recollections of event, externally cued  Distressing recollections of event, not externally cued  Nightmares  Re-living traumatic event  Physiological reactivity to reminders of event  Efforts to avoid reminders of the event  Inability to recall important aspects of the trauma  Feeling of detachment or estrangement from others  Restricted range of positive affect  Restricted range of negative affect  Sense of a foreshortened future  Sleep problems  Irritability  Outbursts of anger  Difficulties concentrating  Hypervigilance  Exaggerated startle response |
| Other anxieties | Hypochondriasis  Social anxiety  Fear of activities in public  Agoraphobia  Animal fears  Fear of injury  Fear of blood/injection  Other specific phobias  Situational anxiety  Free floating anxiety  Selective mutism |
| CD^b^ | Fights more than once per month  Used weapon more than once  Cruel to animals  Stealing without confrontation  Stealing with confrontation  Deliberately started fire  Deliberately damaged others’ property  Broken into house, building or car  Lies at least weekly  Has run away overnight twice or more  Often truant from school |
| ODD | Loses temper  Argues  Defies requests or rules  Deliberately annoys people  Blames others for own mistakes or misbehaviour  Touchy or easily annoyed  Angry and resentful  Spiteful or vindictive |
| Disorder | PCI-derived symptoms included in CFA |
| ADHD-1 | Fails to give close attention to details or makes careless mistakes  Difficulty sustaining attention in tasks or play activities  Does not seem to listen when spoken to  Problems in following through on instructions  Difficulty in organizing tasks and activities  Avoids tasks that require sustained mental effort  Often loses things  Easily distracted  Forgetful in daily activities |
| ADHD-2 | Fidgets or squirms in seat  Leaves seat  Runs about or climbs excessively  Difficulty playing or engaging in leisure activities quietly  ‘On the go’ or ‘driven by a motor’  Talks excessively  Blurts out answers  Difficulty awaiting turn  Interrupts or intrudes on others |
| ASD | Severe impairment in reciprocal social interaction  All-absorbing narrow interest  Imposition of routines and interests  Speech and language problems  Non-verbal communication problems  Motor clumsiness |

Note: *ADHD-1* attention deficit hyperactive disorder (ADHD) attention deficit type; *ADHD-2* ADHD hyperactive and impulsive type; *ASD* autism spectrum disorder; *CAPA* Child and Adolescent Psychiatric Interview; *CD* conduct disorder; *GAD* generalized anxiety disorder; *MDD* major depressive disorder; *ODD* oppositional defiant disorder; *PCI* primary contact interview; *PTSD* posttraumatic stress disorder.

^a^ Panic attacks: The items for ‘paresthesia’ (*n*=0) and ‘chills or hot flushes’ (*n*=0) were excluded in the statistical analysis due to no positive scores.

^b^ Conduct disorder (CD): The items ‘forced someone into sexual activity’ (*n*=0) and ‘cruel to people’ (*n*=2) were excluded due to few cases, thus not fulfilling the requirements for CFA.

Figure S2. Confirmatory factor analysis of reactive attachment disorder (RAD) versus disinhibited social engagement disorder (DSED) in a two-factor and one-factor model, respectively. Standardized factor loadings with standard errors in parenthesis.


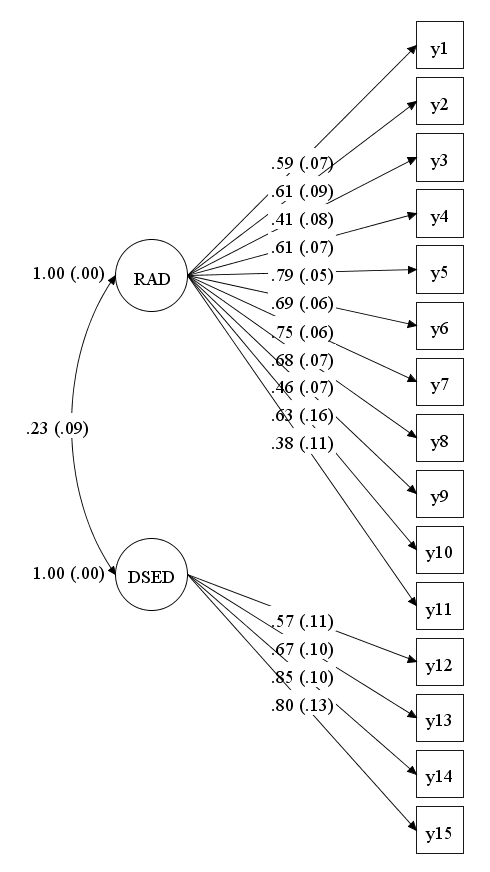

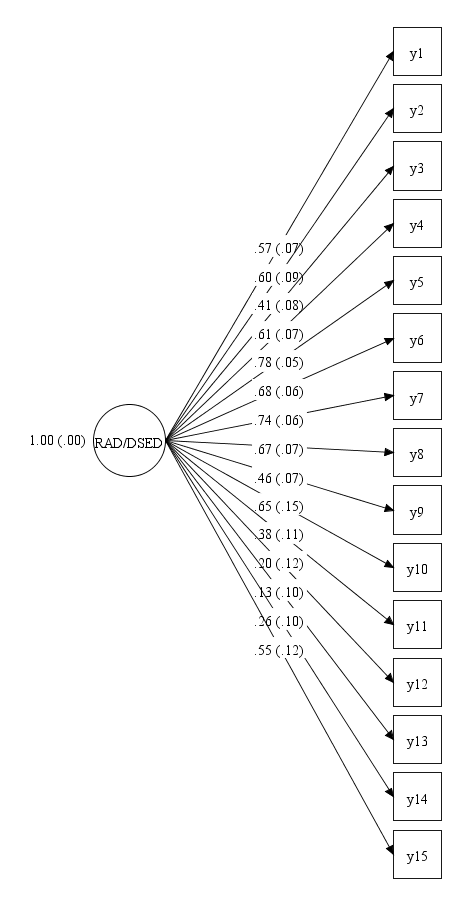


y1 = Inhibition during social interactions
y2 = Lack of interest in family members and peers
y3 = Does not seek comfort when distressed
y4 = Lack of emotional sensitivity
y5 = Difficulty being affectionate
y6 = Avoids physical contact
y7 = Constricted range of facial expression
y8 = Avoids eye contact
y9 = Highly ambivalent and contradictory responses
y10 = Negative reunion responses
y11 = Hypervigilance
y12 = Indiscriminate adult relationships
y13 = Indiscriminate peer relationships
y14 = Indiscriminate willingness to leave with unfamiliar adult
y15 = Minimal checking with caregiver in unfamiliar settings
